# Formaldehyde Fixation Helps Preserve the Proteome State during Single-Cell Proteomics Sample Processing and Analysis

**DOI:** 10.1021/acs.jproteome.4c00656

**Published:** 2025-02-04

**Authors:** Ilaria Piga, Claire Koenig, Maico Lechner, Pierre Sabatier, Jesper V. Olsen

**Affiliations:** † Novo Nordisk Foundation Center for Protein Research, Proteomics Program, Faculty of Health and Medical Sciences, University of Copenhagen, 2200 Copenhagen, Denmark; ‡ Cardio-Thoracic Translational Medicine (CTTM) Lab, Department of Surgical Sciences, Uppsala University, 753 10 Uppsala, Sweden

**Keywords:** single-cell proteomics, formaldehyde fixation, cell-sorting, One-Tip

## Abstract

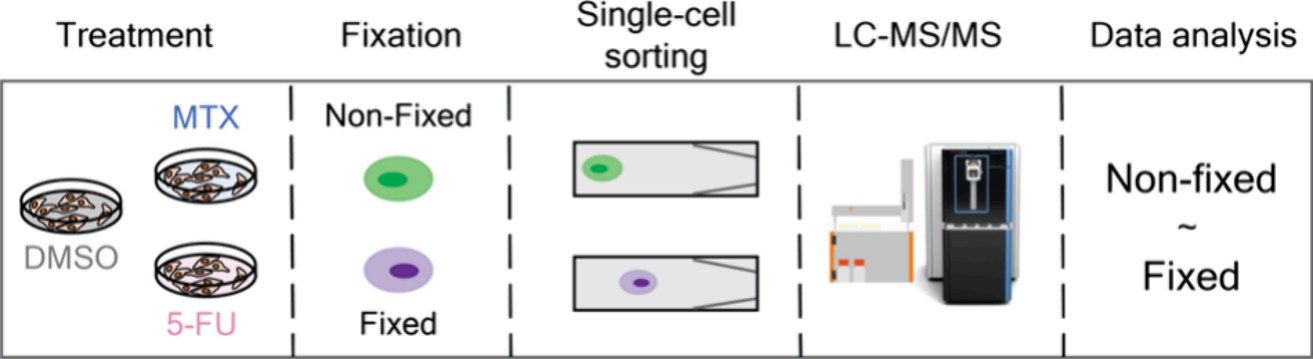

Mass spectrometry-based single-cell proteomics (SCP) is gaining momentum but remains limited to a few laboratories due to the high costs and specialized expertise required. The ability to send samples to specialized core facilities would benefit nonspecialist laboratories and popularize SCP for biological applications. However, no methods have been tested in SCP to “freeze” the proteome state while maintaining cell integrity for transfer between laboratories or prolonged sorting using fluorescence-activated cell sorting (FACS). This study evaluates whether short-term formaldehyde (FA) fixation can maintain the cell states. We demonstrate that short-term FA fixation does not substantially affect protein recovery, even without heating and strong detergents, and maintains analytical depth compared with classical workflows. Fixation also preserves drug-induced specific perturbations of the protein abundance during cell sorting and sample preparation for SCP analysis. Our findings suggest that FA fixation can facilitate SCP by enabling sample shipping and prolonged sorting, potentially democratizing access to SCP technology and expanding its application in biological research, thereby accelerating discoveries in cell biology and personalized medicine.

## Introduction

Single-cell proteomics (SCP) is developing rapidly, and the current analytical depth allows for advanced biological studies.^1−7^ However, while the field was already initiated years ago,^8^ it is not yet widespread in many areas of biology and remains a technology for specialists, and even among expert proteomics laboratories, only a small proportion is fully focused on SCP analyses. This stems both from the expertise required to perform such sensitive analysis but also in large part from costs.^9^ SCP heavily relies on recent advancements in expensive instrumentation encompassing sorters^10^ and systems for sample preparation as well as mass spectrometers. Current SCP methods are limited by the need for immediate sample preparation and analysis, which restricts their broader application and collaboration across laboratories. Thus, state-of-the-art SCP risks remaining accessible only to a minority of laboratories and mostly proteomics-focused laboratories that are able to perform in-house preparation and analysis. Being able to ship cells to collaborators allowing all the steps of preparation while preserving biological information from the samples can contribute extending the reach of SCP. It is possible to sort cells and ship processed samples or cell pellets, but sending samples for sorting is challenging as freezing results in cell damage and protein leakage,^11^ and usually samples are prepared and only shipped for LC-MS/MS analysis as peptides, which then forces laboratories to develop dedicated single-cell proteomics sample preparation pipelines.

Formaldehyde (FA) fixation is traditionally used to preserve the cellular state for extended periods,^12^ allowing for the storage of tissue samples for decades and their manipulation at room temperature. However, removing the cross-link usually requires additional heating steps at high-temperature, strong solubilizing agents, and sonication, which are not directly compatible with sample preparation for high-sensitivity proteomics workflows such as One-Tip^6^ and single-cell analysis,^13,10,14−18^ but necessitate adjustment of the protocols in the best case. In this study, we explored short-term formaldehyde fixation as a way to preserve the in vivo state of protein abundance to allow for prolonged preservation of cell suspensions outside of their culture media for cell sorting and proteomics sample preparation. To assess the effect of fixation on proteome coverage, we first compared bulk preparation of nonfixed and fixed cells using a strong detergent for cell lysis and protein aggregation capture (PAC)-based digestion,^19^ heating the samples at 95 °C followed by sonication. Following this, we applied fixation to a single-cell proteomics label-free workflow,^5,20,10^ which relies on MS-compatible nonionic detergents without high-temperature heating steps, to sort and lyse single cells and digest their proteins for analysis. This analysis showed that results obtained with fixed single cells matched results obtained in nonfixed single cells. We confirmed that biological information on the samples is maintained in bulk as well as at the single-cell level in fixed samples compared to nonfixed controls for samples using treatments with methotrexate (MTX) and 5-fluorouracil (5-FU). Lastly, we investigated the use of fixation for One-Tip analysis^6^ which would extend it to other types of one-pot sample preparation methods^14,16,17,21−25^ for sensitive proteomics using very limited cell numbers.

Overall, our study demonstrates that short-term FA fixation can be used for proteomics analysis without the need for strong detergent or extensive heating/sonication to recover proteins, which could enable shipping of single-cell suspensions between laboratories and extensive FACS sorting for single-cell or sensitive proteomics applications. This would promote the emergence of dedicated single-cell proteomics facilities by allowing people to send samples that could be sorted and processed at the facility without the need to be physically present.

## Experimental Procedures

### Cell Culture

HeLa cells were cultured in DMEM (Gibco, Invitrogen), supplemented with 10% fetal bovine serum and 100 μg/mL penicillin/streptomycin (Invitrogen), at 37 °C, in a humidified incubator with 5% CO_2_. SCC25 were cultured in DMEM/F12 (Gibco, Invitrogen), supplemented with 10% fetal bovine serum and 100 μg/mL penicillin/streptomycin (Invitrogen), at 37 °C, in a humidified incubator with 5% CO_2_.

### Cell Cycle Synchronization with Thymidine and Nocodazole

HeLa cells were synchronized in G1-S overnight with a thymidine block at a concentration of 4 mM (Sigma). Cells were then released from thymidine for 7.5 h (S-G2 phase), and cells were arrested overnight with nocodazole. The next morning, these cells were released for 0.5 h (M phase).

### Cell Treatment with Methotrexate and 5-Fluorouracil

HeLa cells were plated in P6 dishes until they reached 80% confluence. Cells were treated with 6 nM Methotrexate (Selleckem) or with 5 μM 5-fluorouracil (Sigma-Aldrich) or vehicle (DMSO) for 48 h. Subsequently, the cells were detached using trypsin and washed twice with phosphate buffered saline (PBS) from Gibco (Life Technologies) before being resuspended in degassed PBS.

### Fixation

SCC25 and HeLa cells were first detached using trypsin and washed once with PBS. 1%, 2% or 4% formaldehyde (w/v) solution were prepared under a chemical hood by diluting 16% methanol-free formaldehyde (Fisher Scientific) with cold PBS. The cell pellet was then resuspended in the fixing solution and incubated on ice for 20 min.

After fixation, the formaldehyde solution was aspirated under the chemical hood and disposed of in the organic waste container (nonhalogenated and sulfur-free). Cells were washed twice with PBS, with the wash solutions being similarly disposed of in waste group H. Finally, the cell pellet was resuspended in degassed PBS for subsequent use.

### Cell Isolation

**Figure 1:**
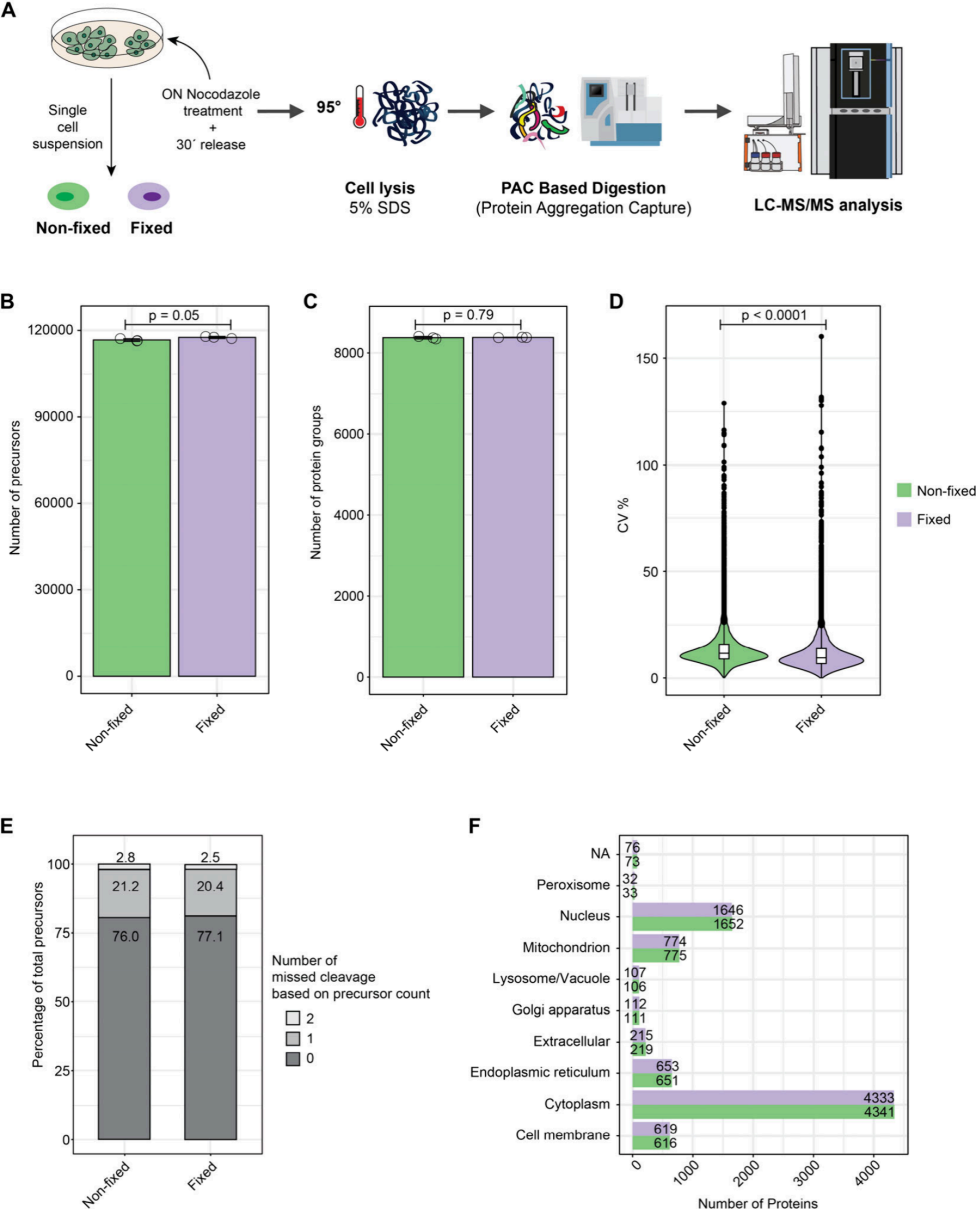
**Effect of fixation on bulk proteomics analysis.** (**A**) Workflow. ON = overnight. (**B**) Number of precursors and (**C**) protein groups identified; (**D**) Violin plot of the coefficient of variation in percentage between replicates; (**E**) Missed cleavage numbers and percentages; (**F**) number of proteins identified in various cellular compartments according to DeePloc 2.0 analysis;^29^ in bulk nonfixed and fixed samples for each treatment. *n* = 3 technical replicates. Error bars represent ± the standard deviation of the mean. The horizontal line in the boxplots represent the median, 25th and 75th percentiles, and whiskers represent measurements to the 5th and 95th percentiles. *P*-values were calculated using a two-sided Welch’s *t*-test.

**Figure 2:**
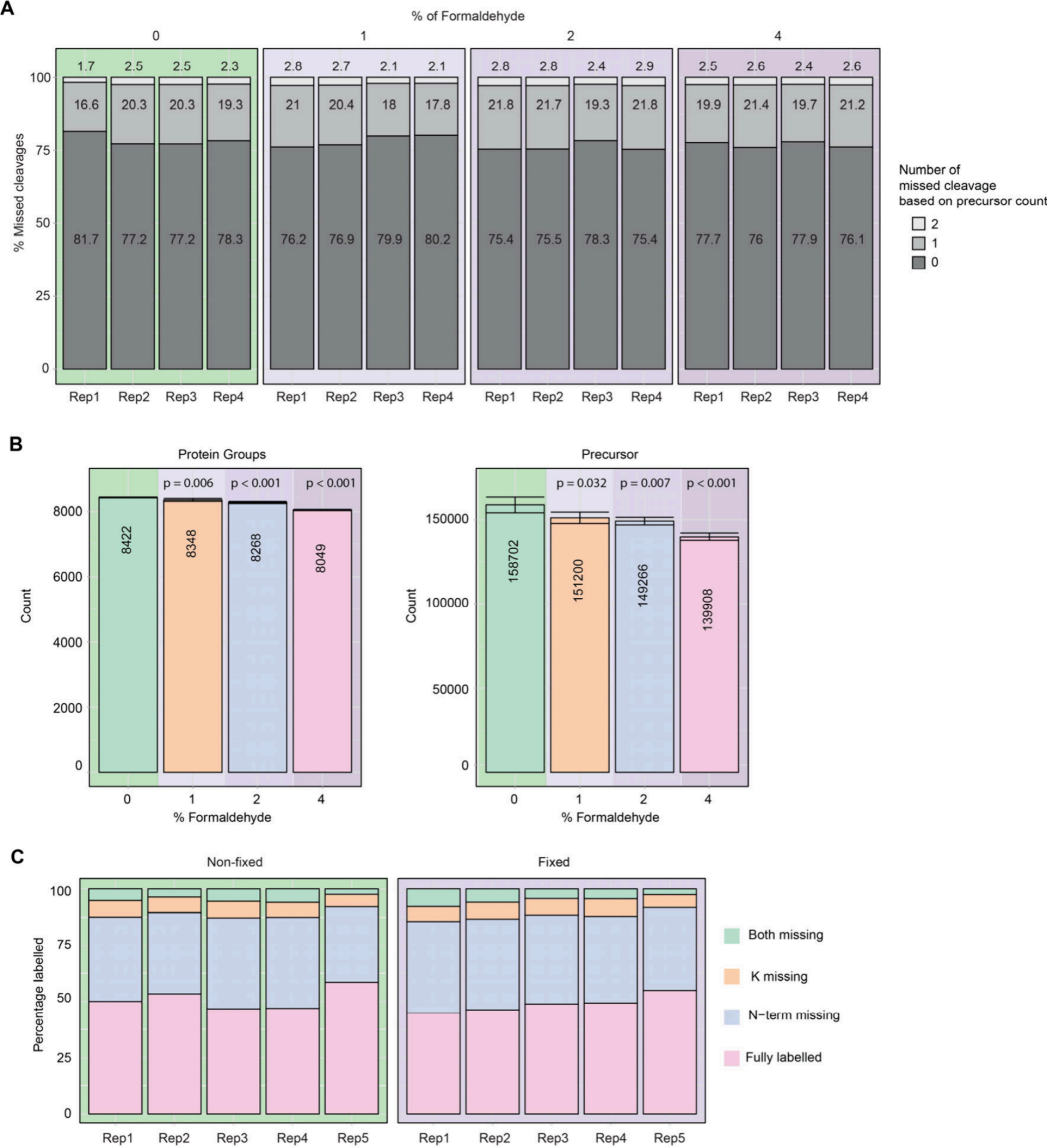
**Effect of FA concentration on missed cleavages, protein and precursor identifications and TMT labeling.** (**A**) Percentages of missed cleavages in One-Tip analysis for cells which were nonfixed or fixed with 1, 2 and 4% FA. *P*-values were calculated using a two-sided Student’s *t*-test for 1, 2 and 4% FA against the nonfixed cells, respectively: 0.85, 0.10, and 0.21 for 0 missed cleavages; 0.54, 0.07, and 0.21 for 1 missed cleavage; 0.89, 0.11, and 0.26 for 2 missed cleavages. *n* = 4 technical replicates for each condition. (**B**) Number of protein groups and precursors identified for each condition. *P*-values were calculated using a two-sided Student’s *t*-test for 1, 2 and 4% FA against the nonfixed cells. *n* = 4 technical replicates for each condition. (**C**) TMT incorporation percentage on lysine and N-terminal. *P*-value between nonfixed and fixed with 2% FA for the missing labels on lysine was 0.30 and calculated using a two-sided Student’s *t*-test. *n* = 5 technical replicates for each condition.

**Figure 3:**
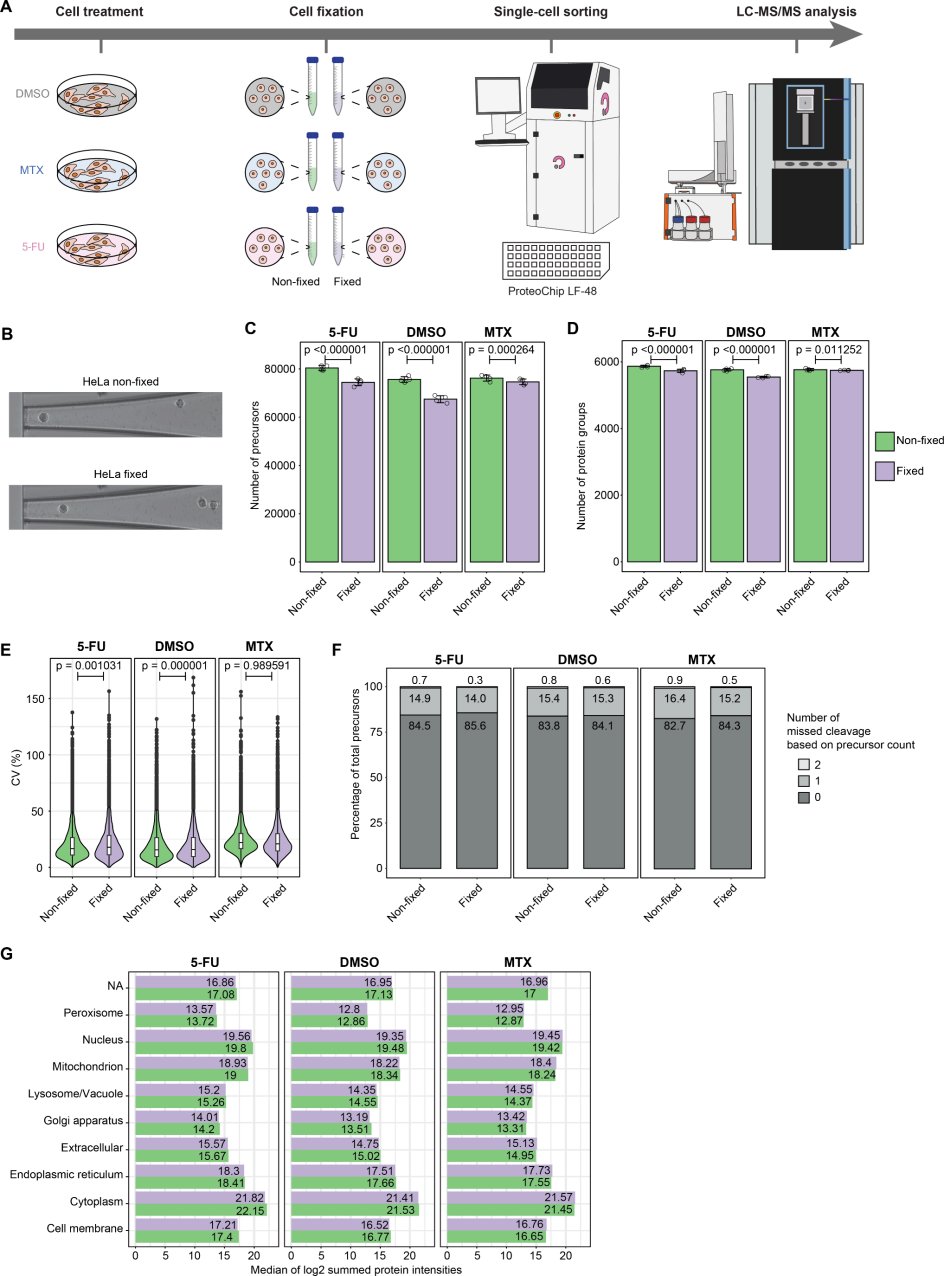
**20 cells and single cell sample preparation and LC-MS/MS analysis of nonfixed and fixed HeLa cells treated with 5-FU and MTX.** (**A**) Workflow. (**B**) Representative pictures of single nonfixed and fixed HeLa cells being dispensed in the cellenONE. (**C**) Number of precursors and (**D**) protein groups identified; (**E**) Violin plots of CVs in percentage between replicates; (**F**) Mean missed cleavage numbers and percentages between replicates; (**G**) log2-transformed summed protein intensities of proteins identified in various cellular compartments according to DeePloc 2.0 analysis; in 20 cells nonfixed and fixed samples for each treatment. *n* = 4 technical replicates. Error bars represent ± the standard deviation of the mean. The horizontal line in the boxplots represent the median, 25th and 75th percentiles and whiskers represent measurements to the 5th and 95th percentiles. *P*-values were calculated using a two-sided Welch’s *t*-test.

**Figure 4:**
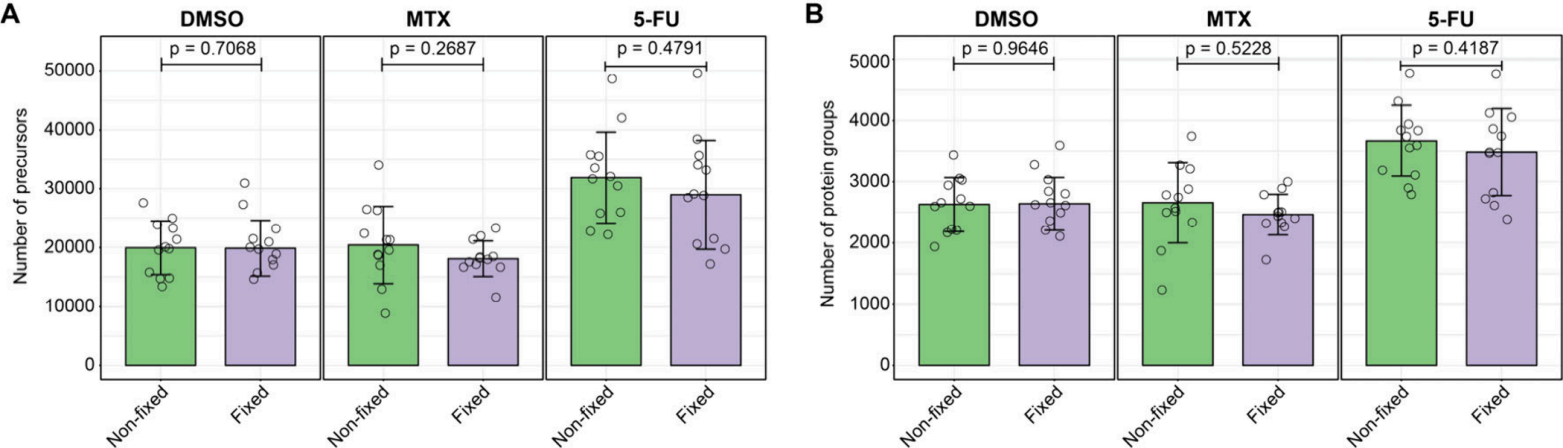
**Number of precursors and protein groups identified in nonfixed and fixed single-cell samples treated with MTX, 5-FU and DMSO controls.** (**A**) Number of precursors. (**B**) Number of protein groups. Error bars represent ± the standard deviation of the mean. *n* = 12 technical replicates. *P*-values were calculated using a two-sided Welch’s *t*-test.

**Figure 5:**
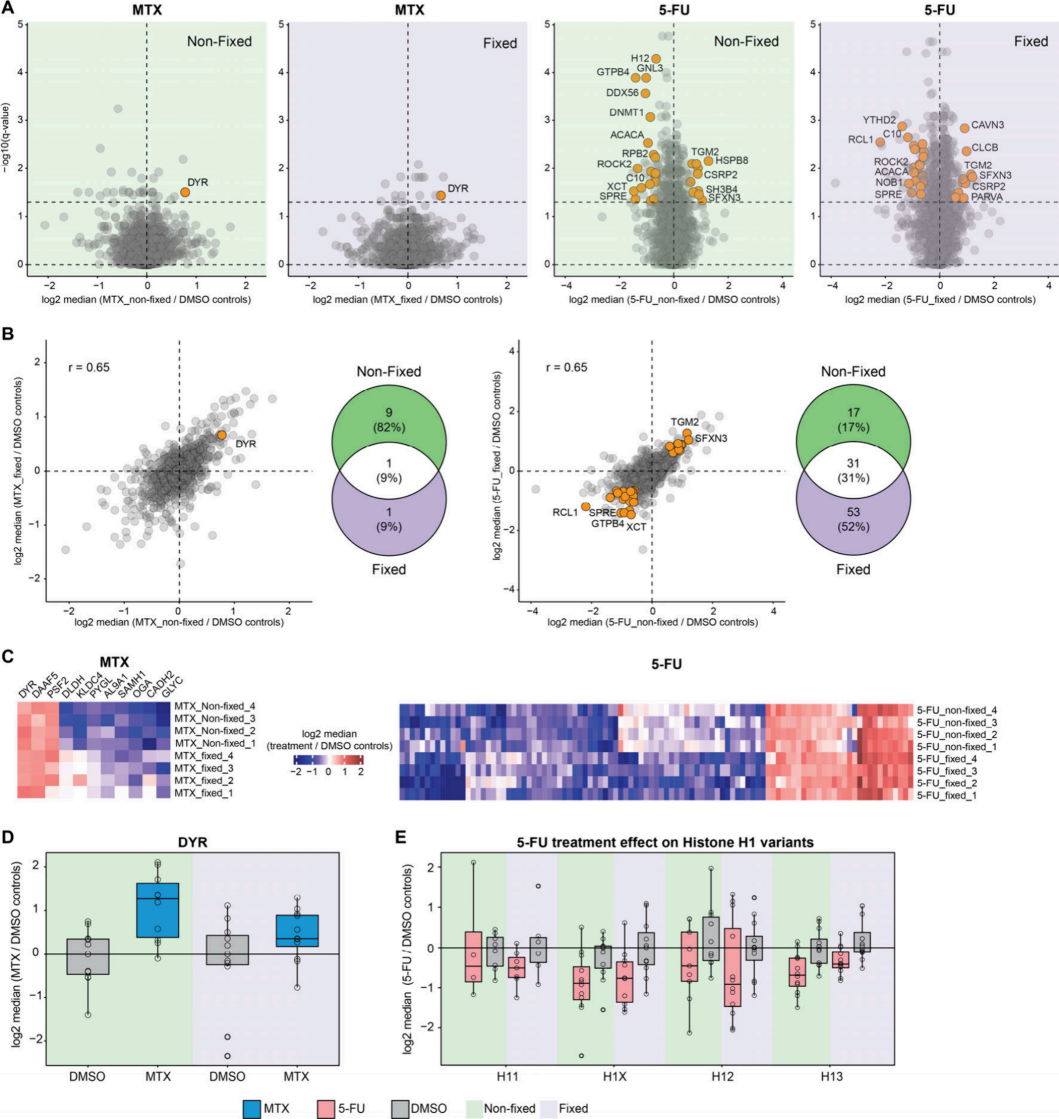
**Effects of MTX and 5-FU treatments on 20 cells and single cells.** (**A**) Volcano plots of the log2 median ratio of each treatment against DMSO controls for nonfixed and fixed 20 cells samples. The shared up- and downregulated proteins between nonfixed and fixed samples are highlighted in orange (*q*-value <0.05 and abs (log2 median ratio) > 0.58). DYR target of MTX and DPYD target of 5-FU are highlighted in red. *q*-values were calculated using a two-sided *t*-test first and then corrected for multiple hypothesis testing using Benjamini-Hochberg procedure. (**B**) 2D plots of the log2 median ratio of each treatment against DMSO controls for nonfixed (*x*-axis) versus fixed (*y*-axis) samples and Venn diagrams representing the shared and nonshared significantly regulated proteins between nonfixed and fixed conditions. Pearson correlations were calculated between nonfixed and fixed conditions. (**C**) Heatmaps representing the log2 ratio of the abundance of significantly regulated proteins in either nonfixed or fixed cells against the median abundance in DMSO controls for each treatment replicate. (**D**) log2 median of MTX-treated single cells against DMSO control for DYR in nonfixed and fixed cells. (**E**) Similar plots but for 5-FU treatment highlighting histone H1 variants. Error bars represent ± the standard deviation of the mean. *n* = 4 technical replicates for 20 cells samples and *n* = 12 technical replicates for single-cell samples, per condition.

**Figure 6:**
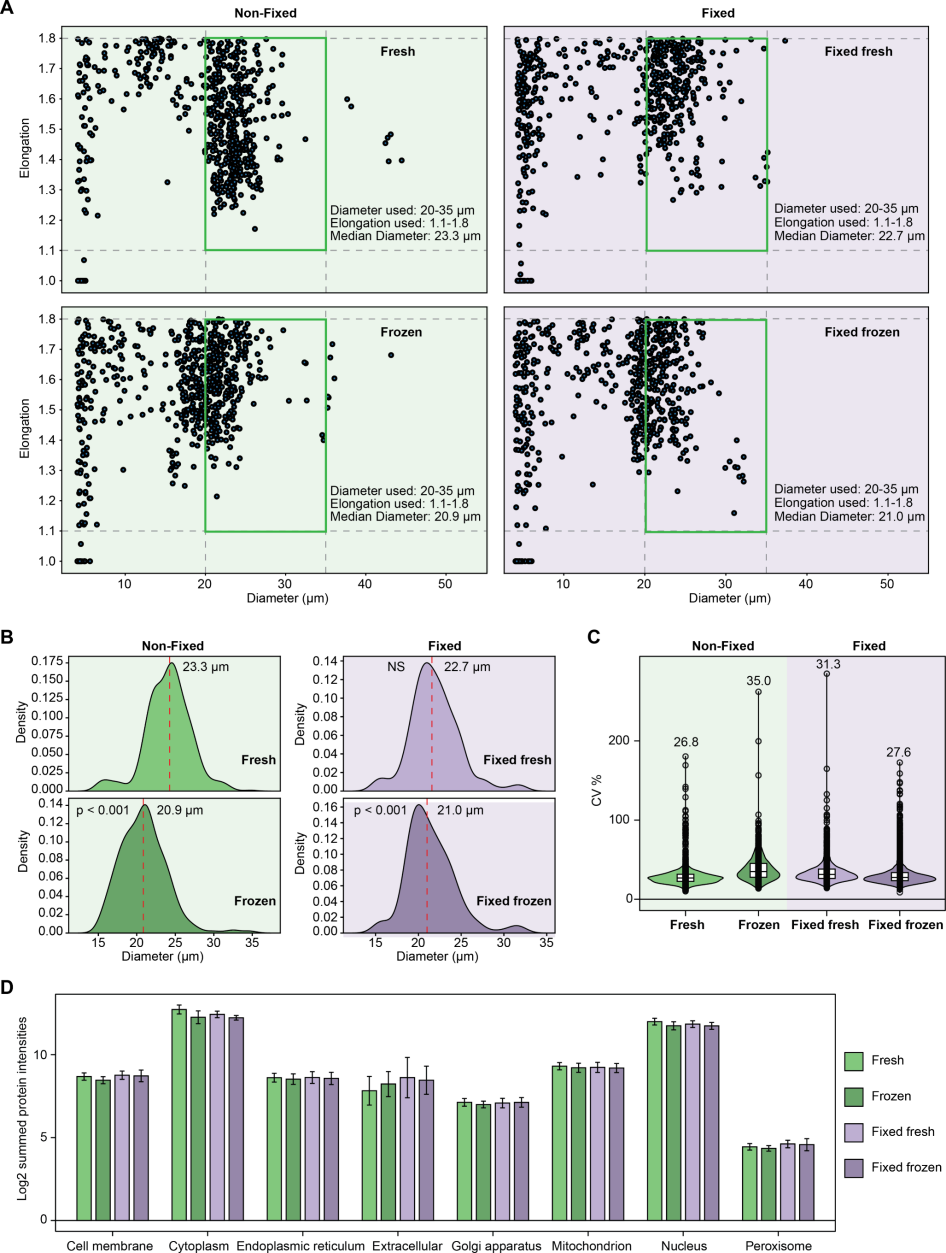
**Effect of fixation and freezing on the sorting parameter and CVs of protein abundances in single cells.** (**A**) Cell diameter and elongation parameters during sorting using cellenONE. The green rectangle represents the selection window which was used for sorting single cells, between 20 and 35 μm diameter and between 1.1 and 1.8 of elongation. (**B**) Distribution of the cells between 15 and 35 μm diameter and between 1.1 and 1.8 of elongation for each condition excluding the size range corresponding to other particles than cells (0 to 15 μm). The median diameter is highlighted in red. *P*-values were calculated using a two-sided student *t*-test and corrected using the Benjamini-Hochberg procedure, and *q*-values <0.05 were considered as significant. NS = Nonsignificant. (**C**) Violin plots of protein abundance CVs among fresh nonfixed cells, nonfixed + frozen cells, fixed cells, and fixed + frozen cells. The median CV is indicated for each violin plot. Only proteins identified in every sample were considered for the analysis. The horizontal line in the boxplots represent the median, 25th and 75th percentiles, and whiskers represent measurements to the 5th and 95th percentiles. (**D**) log2-transformed summed protein intensities of proteins identified in various cellular compartments according to DeePloc 2.0 analysis. Error bars represent ± the standard deviation of the mean. *n* = 14–16 single cells per condition.

The sorting and preparation of HeLa and SCC25 cells concerning the main Figures 1–6 were performed with either LF-48 (according to manufacturer's protocol) or Evo96 proteoCHIPs as described by Ye et al.^6^ using the cellenONE system.

The sorting for HeLa cells were isolated using the cellenONE system. Sample lysis and digestion is performed on the proteoCHIP EVO 96 inside the cellenONE with a temperature control set at 10 °C. Approximately 300 nL of lysis and digestion buffer consisting of 0.2% n-Dodecyl-β-d-Maltoside (DDM) (D4641–500MG, Sigma-Aldrich, Germany), 100 mM triethylammonium bicarbonate (TEAB), 20 ng/μL trypsin, and 10 ng/μL lys-C is dispensed into each well. Cells were sorted into the wells based on a diameter range of 20–35 μm and an elongation factor between 1.1 and 1.8. The proteoCHIP is then incubated within the cellenONE at 50 °C with 85% relative humidity for 1.5 h. The wells were hydrated by continuously dispensing water on top of the lysis buffer throughout the incubation to prevent evaporation. After incubation, the temperature is reduced to 20 °C, after which 4 μL of 0.1% formic acid was manually added to each well. Evotips were prepared according to the manufacturer’s instructions until the sample loading step, where 16 μL of 0.1% formic acid is added to each tip, and the proteoCHIP is inverted on top of the Evotips and centrifuged at 800*g* for 60 s. Then the Evotips were processed again following the manufacturer’s protocol.

### One-Tip Analysis

Before starting the One-Tip protocol, cell concentration was determined using a cell counter and adjusted to reach a final concentration of either 200 or 600 cells/μL. One-Tip preparation was performed as previously described^6^ Evotips were washed with 20 μL of acetonitrile (ACN) and centrifuged at 800*g* for 60 s. Evotips were then soaked in 1-propanol until they turned pale white and equilibrated with 20 μL of Solvent A (0.1% formic acid in water) and centrifuged at 800*g* for 60 s. 5 μL of lysis/digestion buffer and 5 μL of cells were loaded into the Evotips (either 1000 or 3000 cells in total). Lysis and digestion buffer contains 0.2% DDM, 100 mM TEAB, 20 ng/μL Trypsin, and 10 ng/μL Lys-C. Evotips containing digestion buffer and cells mixture were briefly centrifuged at 50*g* to mix the buffer and cells and prevent the formation of air bubbles. Water was added inside the Evotip box to the level of the C_18_ resin in the Evotips and incubated for 3 h at 37 °C.

### Lysis and Digestion

For bulk experiments, cells were lysed with 80 μL of boiling lysis buffer (5% SDS, 100 mM Tris pH 8.5, 5 mM TCEP, and 10 mM CAA). The lysates were incubated at 95 °C for 10 min with mixing (500 rpm) and sonicated using a Branson probe sonicator for 1 min using a 1 s pulse and 30% amplitude.

Protein concentration was measured by the BCA assay. Protein digestion was performed according to the Protein Aggregation Capture (PAC)-based digestion^26^ on a KingFisher Flex System (Thermo Scientific)^27^ with magnetic hydroxyl beads (ReSyn Biosciences). KingFisher deep-well plates were prepared for washing steps, containing 1 mL of 95% ACN or 70% Ethanol (EtOH). For each sample, 200 μL of digestion solution (50 mM triethylammonium bicarbonate) containing Lys-C and Trypsin at an enzyme-to-substrate ratio of 1:500 and 1:250, respectively, were prepared and transferred to KingFisher plates. Samples were mixed with ACN to achieve a final volume percentage of 70%. The storage solution from the hydroxyl beads was replaced with 70% ACN. Finally, beads were added to the samples at a protein bead ratio of 1:2. Protein aggregation was carried out in two steps of 1 min of mixing at medium speed, followed by a 10 min pause each. Sequential washes were performed in 2.5 min at a slow speed without releasing the beads from the magnet. Digestion was set to 100 cycles of agitation for 45 s and a pausing of 6 min overnight at 37 °C. Protease activity was quenched by acidification with trifluoroacetic acid (TFA) to a final volume percentage of 1%.

### TMT-Labeling

1000 cells were digested using the One-Tip protocol and eluted with 20 μL of 50% ACN. HEPES (1M, pH 8) was added to reach a final concentration of 100 mM. TMTproZero was added to the peptides at a ratio of 1:2 (peptide:TMT (w/w)). The samples were incubated for 1 h at room temperature and acidified to reach 1% TFA final, and the ACN was evaporated. The peptides were resuspended in 20 μL of buffer A and loaded on Evotips.

### LC-MS/MS

LC-MS/MS analysis was performed on an Orbitrap Astral MS using Thermo Fisher Scientific Tune software (version 0.4 or higher) using narrow-window (n)DIA^28^ coupled to an Evosep One system (EvoSep Biosystems). Single cell and One-Tip samples were analyzed on a 40 samples per day (SPD) (31 min) gradient using a commercial analytical column (Aurora Elite TS, IonOpticks) interfaced online using an EASY-Spray source. Proteome bulk and TMT labeled peptides were separated on an 80 SPD gradient. The Orbitrap Astral MS was operated at a full MS resolution of 240,000 with a full scan range of 380–980 *m*/*z* when stated. The full MS AGC was set to 500%. MS/MS scans were recorded with 4Th isolation window, 6 ms maximum ion injection time (IT) for cellenONE-sorted cells, and One- Tip samples. MS/MS scanning range from 380–980 *m*/*z* were used. The isolated ions were fragmented using HCD with 25% NCE. TMT-labeled peptides were analyzed using an Orbitrap-Orbitrap-based Top 10 DDA method, where MS1 resolution was set at 60,000 over a mass range from 350 to 1400 *m*/*z*. MS2 resolution was set at 45,000 with a NCE of 35%.

### MS Data Analysis

Raw files were analyzed in Spectronaut v18 (for all the initial experiments including Nocodazole treatment, Metotraxate and 5-Fluoracil treatment) and 19 (for the analyses presented in Supplementary Figures) (Biognosys) with a spectral library-free approach (directDIA+) using the human protein reference database (Uniprot 2022 release, 20,588 sequences) and complemented with common contaminants (246 sequences). Note, as the protocol does not involve reduction and alkylation, database searches were performed with free cysteine sulfhydryls, and hence cysteine carbamylation was not set as a fixed modification, whereas methionine oxidation and protein N-termini acetylation were set as variable modifications. Carbamylation was included as a fixed modification for the PAC digested samples. Precursor filtering was set to perform based on q-values, and cross run normalization was unchecked. MS2 quantification was used.

DDA data was searched on MQ 2.0.1.0, allowing up to 2 missed cleavages, carbamylation was removed from fixed modifications, and TMTproZero was added as a variable modification on the N-terminal and on lysine residues.

### Data Availability

The mass spectrometry proteomics data generated in this study have been deposited to ProteomeXchange Consortium (http://proteomecentral.proteomexchange.org) via the PRIDE partner repository with data set identifier PXD054445, which contains the raw data used to generate Figure 1, Figure 3, Figure 4, Figure 5, Figure S1, Figure S2, Figure S3, and PXD056327, which contains the raw data used to generate Figure 2 and Figure S4.

## Results

### Effect of Fixation on Bulk Sample Preparation Using Strong Detergent and Lysis Methods

To investigate the effect of short-term FA fixation on LC-MS/MS measurements of protein abundance, we synchronized HeLa cells in different cell cycle stages by initially arresting them in the G1/S phase using thymidine followed by nocodazole treatment and the removal of the drug leading to subsequent changes in protein abundance. We then performed bulk analysis with half of the samples being fixed with FA and the other half nonfixed (Figure 1A). To avoid variation due to the time difference in sample preparation, monitor the direct effect of drug treatment, and allow an unbiased comparison with fixed samples, the nonfixed samples were directly lysed and processed, while the fixed samples were fixed first and then processed in parallel with the nonfixed samples. The bulk analysis was performed with a PAC sample preparation protocol including 5% of the strong detergent sodium dodecyl sulfate (SDS) and a heating step at 95 °C followed by sonication prior to protein aggregation on magnetic microbeads and on-bead digestion with Lys-C and trypsin. The heating step should effectively reverse the FA cross-links, and thus we did not expect large differences between fixed and nonfixed samples, and if any, they would be due to the fixating agent. All samples were analyzed by liquid chromatography tandem mass spectrometry (LC-MS/MS) using the Evosep One LC connected to the Orbitrap Astral mass spectrometer operated in narrow window data-independent acquisition (nDIA).^28^ There was indeed no significant difference (*p* < 0.05) in the number of precursors and proteins quantified (Figures 1B and 1C). Since FA cross-links proteins potentially covering enzyme cutting sites, we assumed that the miss-cleavage rate could be higher in fixed samples, which was not the case here (Figure 1D). The samples showed also a high correlation of protein abundances between similar conditions (Figure S1). The coefficient of variation (CV) between replicates was significantly lower in fixed compared to nonfixed samples, but by a small margin, which could reflect variation during sample preparation rather than the effect of the fixation (Figure 1D). Lastly, there was no difference in the number of proteins identified at various cellular localization between nonfixed and fixed samples (Figure 1F). This comes as little surprise, as this protocol follows similar steps (i.e., strong detergent, heating, and sonication) to the standard protocols used for analyzing fixed samples. Therefore, it should allow for a high recovery of proteins. These results indicate that short-term formaldehyde fixation does not compromise protein recovery or analysis depth, making it a viable option for SCP workflows.

### Effect of the Percentage of FA Fixation on Missed Cleavages, Protein and Precursors Identification and TMT Labeling

Since our PAC protocol relies on a strong detergent, high temperature heating, and sonication, which contributes to breaking down the cross-links created by FA, we then tested single-cell and sensitive proteomics protocols that employ the mild detergent N-dodecyl β-d-maltoside (DDM) and no strong cell lysis method (see Experimental Procedures). The use of mild detergents in SC and sensitive proteomics workflows is crucial, as it ensures compatibility with downstream mass spectrometry analysis without compromising cell integrity. Since the concentration of FA could influence peptide recovery, we tested FA concentrations from 0 to 4% on HeLa cells using One-Tip analysis,^6^ which relies on a similar lysis and digestion buffer as for SCP analysis. We do not observe a significant increase in missed cleavages with any of the FA concentration, compared to nonfixed cells (Figure 2A). However, the number of proteins and precursors identified exhibited a concentration-dependent decrease, which was not sharply marked at 1 and 2% FA compared to nonfixed cells, but was significantly lower in 4% FA by around 400 proteins and 19 000 precursors (Figure 2B). To confirm the applicability of 2% FA fixation, we analyzed nonfixed cells and cells fixed with 2% FA using One-Tip, there were no significant differences in peptide (Figure S2A) and protein numbers (Figure S2B). The correlation of protein relative abundance between replicates and between nonfixed and fixed cells were also very high, highlighting the reproducibility of the analysis (Figure S2C). Therefore, we selected 2% FA for the rest of the study since it provides more identifications while reducing the use of toxic material and potential cross-linking artifacts compared to 4% FA.

Lastly, we investigated the effect of fixation on sample multiplexing, since it is the most popular way of performing SCP analysis. We prepared samples using One-Tip, eluted the peptides, and labeled them with a tandem mass tag (TMT) proZero. Fixation did not appear to decrease TMT labeling efficiency (Figure 2C) since the percentage of labeled lysines was not significantly different between fixed and nonfixed samples. This shows that fixation can be applied to multiplexed workflow using TMT and likely, by extension, to other amine and lysine-based labeling reagents such as dimethyl^30^ and mTRAQ labeling for plex-DIA.^14^ Of note, the labeling efficiency displayed here is lower than what could be expected using an optimized protocol for sensitive analysis. The low labeling efficiency allows for a valid comparison between fixed and nonfixed samples, however, for broader biological applications it would lead to increased sample complexity.

### Impact of FA Fixation on Few-Cells, Single-Cell Sorting, and Sample Preparations Using a Dedicated SCP Workflow

In parallel to the bulk analysis of arrested cells, we produced a single-cell suspension of each treatment, and here again, half was fixed using FA and the other half was left in PBS. We also evaluated treatments with the anticancer chemotherapeutic drugs methotrexate (MTX) and 5-fluorouracil (5-FU) (Figure 3A). For SCP analyses, cells were sorted and prepared as few-cell preparations (20 cells) and individual single cells using the cellenONE platform for single-cell isolation and nanoliter dispensing. This experiment replicated a typical sorting experiment, which would happen in a laboratory specialized in single-cell proteomics analysis. For this particular analysis, cells were sorted into the LF-48 proteoCHIP. We did not observe significant alteration of cell morphology of fixed cells compared to nonfixed cells during the sorting using the cellenONE (Figures 3B, 6A and 6B) and the sorting proceeded evenly for both types of samples. Nonfixed cells exhibited significantly higher peptide and slightly higher protein numbers (3684, 2680, 2686 for nonfixed cells against 3513, 2766, 2518 for fixed cells, respectively) across all three treatments compared to their fixed counterparts apart for the MTX treatment (Figures 3C and 3D); the CVs between replicates showed similar pattern (Figure 3E), while the number of missed cleavages were on similar levels overall (82.7 to 85.6% of normally cleaved precursors) (Figure 3F), and the summed intensities of proteins quantified in each subcellular compartment were on similar level in nonfixed and fixed samples (Figure 3G), as in the bulk analysis using PAC.

For single-cell samples, there were no significant differences in precursors and protein numbers between fixed and nonfixed samples (Figures 4A and 4B). Interestingly, the 5-FU-treated samples had many more precursors and proteins identified, which likely stem from the effect of the treatment. There was no significant difference in arrested and then released cells for single-cell samples (Figures S3A and S3B). Since the depth was not different in the single-cell samples from MTX, DMSO and 5-FU and in the released cells between nonfixed and fixed, the difference observed for the 20 cells in 5-FU, DMSO and MTX treatments could be a technical artifact. These results were surprising since the preparation employed in our label-free single-cell proteomics analysis relies, as most single-cell sample preparations, on 0.2% DDM, which is much weaker than SDS for solubilizing proteins and no strong heating nor sonication is used to help solubilize proteins. Since the FA fixation did not alter the analytical depth or protein recovery, this highlights the potential of fixation for single-cell proteomics sample preparation and analysis, where analytical depth is key.

### Formaldehyde Fixation Maintains Specific Changes in Protein Abundance upon Drug Perturbation

To verify that specific protein abundance changes are also maintained with fixation after cell sorting and sample preparation for single-cell or sensitive proteomics analysis, we investigated proteome variation in the MTX and 5-FU-treated 20 cells and single-cell samples. We compared the samples treated with MTX and 5-FU to their DMSO control by using statistical analysis for each condition and treatment (Figure 5A). The main target of MTX is dihydrofolate reductase (DYR),^31^ which is upregulated upon treatment,^32−34^ and we observe significant upregulation of DYR in 20 cells samples both in fixed and nonfixed conditions (Figure 5A). In addition, DYR was the only significantly regulated protein shared between nonfixed and fixed cells (Figures 5A and 5B). None of the targets of 5-FU were significantly regulated in the 20 cell samples both in fixed and nonfixed samples, but histone H1 variants were among the most significantly downregulated proteins (Figures 5A and 5B). The Pearson correlation of the ratios of treatment versus DMSO controls for nonfixed compared to fixed condition was 0.65 for both MTX and 5-FU (Figures 6A–C), showing that the information on protein abundance changes in the treatments is largely maintained between fixed and nonfixed samples even after sorting with the cellenONE.

Next, we analyzed the single-cell proteomics data of the MTX and 5-FU treatments. We observe similar behavior for proteins that were detected as up- or downregulated in the analysis of 20 cells. Remarkably, there are noticeable variations in the abundance of those proteins between single cells of a similar condition, likely due to cell variation. Among proteins which were significantly up or downregulated in the 20 cell samples analyses of MTX, DYR also showed an upregulated trend on average in single cells, both in fixed and nonfixed cells (Figure 5D). For 5-FU treatment, the main feature of 5-FU treatment in the 20 cells analysis was the downregulation of histone H1 variants, which was also the case in single cells (Figures 5E). The analytical depth was, as expected, greatly reduced in single cells compared to 20 cells analysis resulting in many missing values in the single-cell samples. Nevertheless, we detect a similar direction for the abundance of proteins which were also detected in most of the single cells when compared to DMSO control, demonstrating that fixation can be used in single-cell proteomics analysis, and it preserves information about changes of protein abundances in treated samples.

Lastly, we analyzed MTX and control treated samples fixed with various concentrations of FA. DYR fold change is similar to nonfixed cells until up to 2% FA, but 4% FA significantly decreases the FC against control, further demonstrating that FA concentration higher than 2% can alter cells’ proteomes (Figure S4).

### Effect of Freezing on Nonfixed and Fixed Cells

Finally, we investigated the effect of freezing the nonfixed and fixed samples, which could further help to preserve fixed cells for long-term storage, as frozen nonfixed cells are prone to cell leakage impacting single-cell preparation.^11^ Both nonfixed and fixed samples showed significant alteration of cell morphology in comparison to freshly prepared nonfixed cells, which was particularly apparent in the distribution of cell diameter from measurements with the cellenONE (Figures 6A and 6B). Nevertheless, we sorted, prepared, and analyzed single HeLa cells using similar diameter and elongation thresholds as for fresh HeLa cells (20–35 μm diameter and 1.1–1.8 elongation) to assess whether it also affected the proteome of the cells. Nonfixed frozen cells exhibited the highest CVs of protein abundance compared to fresh cells with a median of 35.0% and 26.8%, respectively. This suggests that their proteome was affected by the freezing process even when selecting cells of the same size as in the fresh cells group (Figure 6C). Freshly prepared cells had higher summed protein intensities from the cytoplasm and nucleus than the other conditions (Figure 6D). Since fixed, frozen cells showed lower CVs than fixed cells which were directly processed, this could suggest that their proteome remained less affected by the freezing process but should be further investigated.

## Discussion

We have investigated fixation as a way to maintain the cell state in cell suspensions during a sorting period outside of their culture medium. HeLa and SCC25 cell lines were chosen due to their well-characterized proteomes and relevance in cancer research, making them ideal models for this study. We did not observe any significant difference in SCP data between FA fixed and nonfixed cell preparations. This could potentially enable long FACS sorting and transport between laboratories without the need to perform sample preparation in-house first. The lysis and digestion of single-cell and few-cell samples appear unaffected by FA fixation, likely due to the large excess of enzymes and detergent relative to the protein content, facilitating rapid and efficient protein digestion. Additionally, we have not investigated the effect of prolonged storage (more than a week) at 4 °C. This needs to be tested with samples which have stayed fixed for a longer period leading to more cross-links and potentially gradual deterioration of the cells.

Beyond facilitating cell shipping, fixation offers the possibility of monitoring intracellular markers for single-cell sorting, as they require membrane permeabilization, which is incompatible with classical SCP sample preparation. FA fixation enables the use of permeabilization. This would also enable the tagging of intracellular markers for FACS sorting, which are currently limited to surface markers for SCP analysis.

Fixation would also facilitate a presorting with FACS and then sample preparation using a dedicated sample preparation instrument such as the cellenONE, potentially enabling higher analytical depth for FACS-sorted samples and increasing the versatility of SCP workflows. In summary, lengthier and multistep protocols could be designed with fewer concerns about proteome changes during the sample preparation period.

Whether fixation for a long duration truly manages to freeze the proteome changes should be investigated in the future as well as the ability to freeze the PTM landscape of cells for dynamic PTMs such as phosphorylation during receptor stimulation. Nevertheless, this study offers new directions for development of protocols allowing the preservation of cell state for prolonged storage and subsequent single-cell sorting and preparation for proteomics analysis. Future studies should explore the long-term stability of fixed samples and the preservation of post-translational modifications to fully understand the potential and limitations of FA fixation in SCP.

Another important question was assessing the effect of freezing fixed samples in comparison to nonfixed cells and based on our observations, fixation does not prevent the negative effects of freezing on cell morphology. However, cells selected in the same size range as fresh cells showed similar CVs, suggesting that their proteome was better preserved in comparison with frozen, nonfixed cells which showed the largest CVs among the tested conditions. However, this should be studied in more detail to confirm this observation.

Overall, this study demonstrates that short-term FA fixation is a viable method to preserve cell proteomes for SCP, potentially democratizing access to this advanced technology and broadening its application in biological research

## Supporting Information Available

**Figure S1.** Correlation of protein relative abundances in PAC-digested nonfixed and fixed samples. **Figure S2.** Comparison of nonfixed and fixed samples after One-Tip analysis. **Figure S3.** Number of protein and peptide identified in single Hela arrested cells. **Figure S4.** Effect of the percentage of FA during fixation on the fold change of DYR, MTX’s target. (PDF)
