# Supplementary material for: Formaldehyde Fixation Helps Preserve the Proteome State during Single-Cell Proteomics Sample Processing and Analysis
Source: J Proteome Res. 2025 Feb 4;24(4):1624–35. doi: 10.1021/acs.jproteome.4c00656 (PMC11977542; doi:10.1021/acs.jproteome.4c00656)
Supplement: Supplementary file 1 — pr4c00656_si_001.pdf [file pr4c00656_si_001.pdf]

SUPPORTING INFORMATION

Formaldehyde fixation helps preserve the proteome state during single-cell proteomics sample processing and analysis

Ilaria Piga[1], Claire Koenig[1], Maico Lechner[1], Pierre Sabatier[1,2,*], Jesper V. Olsen[1,*]

[1]Novo Nordisk Foundation Center for Protein Research, Proteomics Program, Faculty of Health and Medical Sciences, University of Copenhagen, 2200 Copenhagen, Denmark

[2]Cardio-Thoracic Translational Medicine (CTTM) Lab, Department of Surgical Sciences, Uppsala University, SE-751 05 Uppsala, Sweden

*Corresponding authors:

Pierre Sabatier (PS): pierre.sabatier@uu.se

Jesper V. Olsen (JVO): jesper.olsen@cpr.ku.dk.

Table of contents:

Figure S1. Correlation of protein relative abundances in PAC-digested non-fixed and fixed samples.

Figure S2. Comparison of non-fixed and fixed samples after One-Tip analysis.

Figure S3. Number of protein and peptide identified in single Hela arrested cells.

Figure S4. Effect of the percentage of FA during fixation on the fold change of DYR, MTX's target.

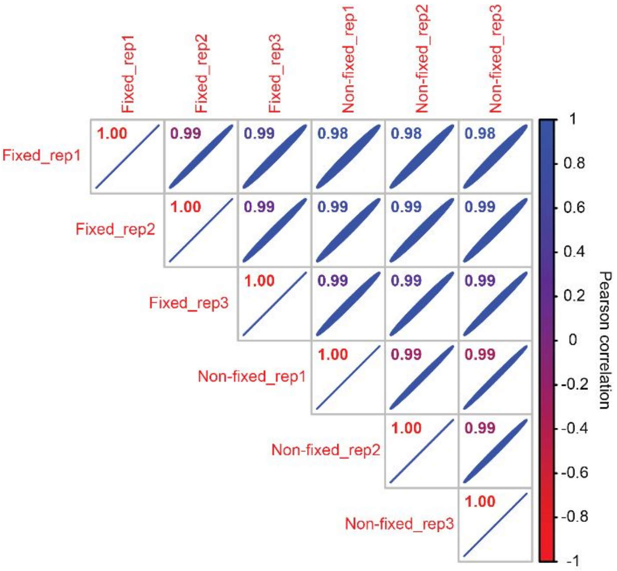

Figure S1. Correlation of protein relative abundances in PAC-digested non-fixed and fixed samples. Pearson correlation of the protein relative abundance between each replicate of PAC-digested non-fixed and fixed samples. n = 3 technical replicates.

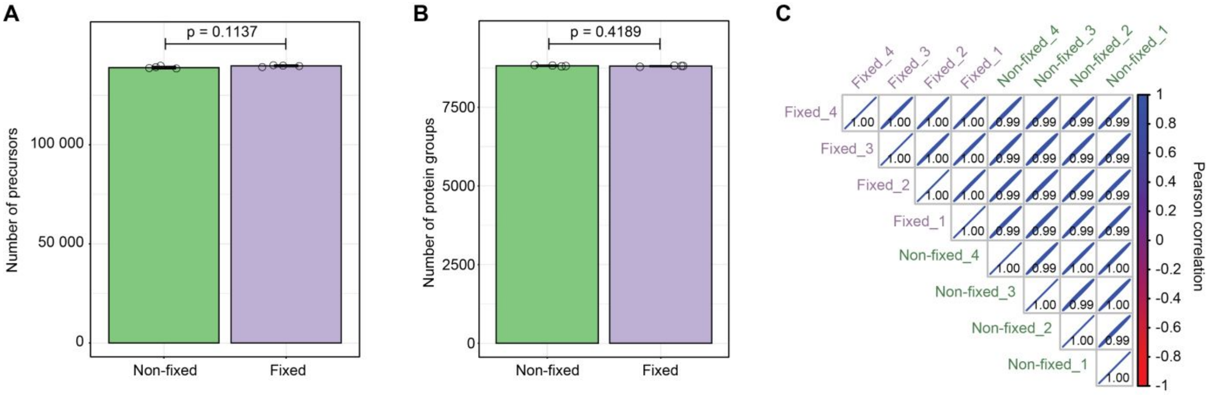

Figure S2. Comparison of non-fixed and fixed samples after One-Tip analysis. (**A**) Number of precursors and (**B**) proteins identified in non-fixed and fixed samples. Error bars represent ± the standard deviation of the mean. n=4 technical replicates. P-values were calculated using a two-sided Welch's *t*-test. (**C**) Pearson correlation of protein relative abundances between replicates.

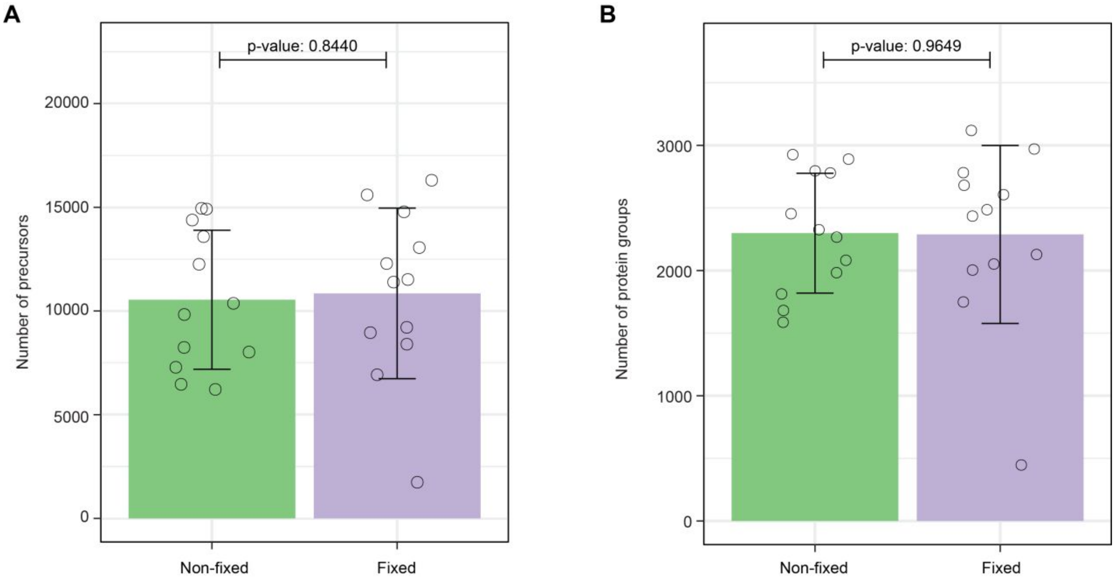

Figure S3. Number of protein and precursors identified in single Hela arrested cells. (**A**) Precursor and (**B**) protein numbers in non-fixed and fixed single cells prepared with the CellenONE. n = 12 non-fixed and n = 11 fixed cells. P-values were calculated using a two-sided Welch's *t*-test.

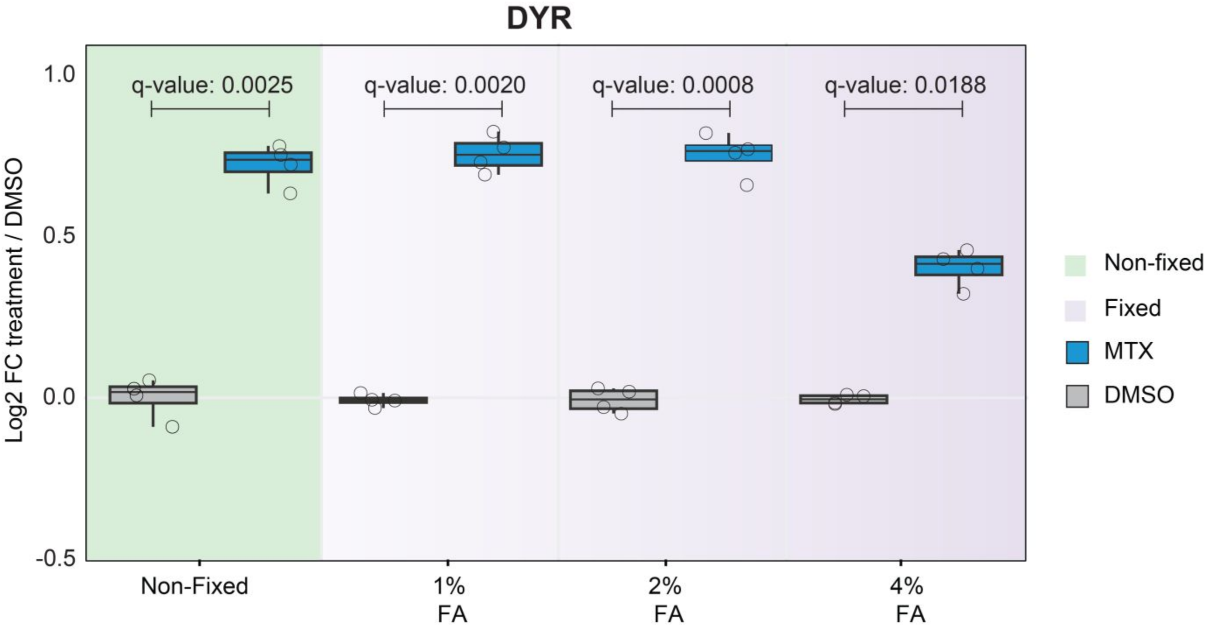

Figure S4. Effect of the percentage of FA during fixation on the fold change of DYR, MTX's target. (**A**) Fold change between cells fixed with 1, 2 and 4% FA against non-fixed cells. q-values were calculated using the limma R package including a correction for multiple comparison.
